# Design, Simulation and Experimental Study of the Linear Magnetic Microactuator

**DOI:** 10.3390/mi9090454

**Published:** 2018-09-11

**Authors:** Hanlin Feng, Xiaodan Miao, Zhuoqing Yang

**Affiliations:** 1 College of Mechanical Engineering, Shanghai University of Engineering Science, Shanghai 201620, China; m010216123@sues.edu.cn; 2 National Key Laboratory of Science and Technology on Micro/Nano Fabrication, School of Electronics Information and Electrical Engineering, Shanghai Jiao Tong University, Shanghai 200240, China; yzhuoqing@sjtu.edu.cn

**Keywords:** MEMS, microactuator, magnetic

## Abstract

This paper reports the design, simulation and experimental study of a linear magnetic microactuator for portable electronic equipment and microsatellite high resolution remote sensing technology. The linear magnetic microactuator consists of a planar microcoil, a supporter and a microspring. Its bistable mechanism can be kept without current by external permanent magnetic force, and can be switched by the bidirectional electromagnetic force. The linearization and threshold of the bistable mechanism was optimized by topology structure design of the microspring. The linear microactuator was then fabricated based on non-silicon technology and the prototype was tested. The testing results indicated that the bistable mechanism was realized with a fast response of 0.96 ms, which verified the simulation and analysis.

## 1. Introduction

“Microactuator” generally refers to a driver with small size, high positioning accuracy and low energy consumption, and is an important part of microelectromechanical systems (MEMS) [1]. Its main function is to realize the transformation and output force or displacement (including displacement and angle), which forms the operation and power for MEMS devices [2]. With the development of portable electronic equipment and microsatellite high resolution remote sensing technology, linear microactuators with fast response and high precision characteristics have been attracting more attention [3,4,5,6]. In recent years, the experimental study and modeling of the voice coil motors (VCM) actuator [3,4] and permanent rotatory linear actuator [7,8] have been presented. However, the VCM actuator comprises two or more permanent magnets, a yoke, a fixed base, a moving part and a coil. These parts were fabricated separately, and then were assembled. The componentry was complex, and as a result, the precision of the actuator was affected, as well as its volume. Closed-loop control was used to improve the precision control of the MEMS microactuator [9,10,11,12]. Sensors were used in the control system, but this lead to increased complexity of the system, and the system will be influenced by environment. Since the displacement of the microactuator is always limited in micrometers, the sensor precision may have a negative influence on the precision control. In addition, the electrostatic, electrothermal and piezoelectric actuators were also studied for high precision control [13,14]. However, the driving voltage of the electrostatic microactuator is above 12 V, which is not compatible with integrated circuit (IC) technology. The electrothermal miroactuator usually responds at a slow speed, and the displacement of the piezoelectric microactuator is limited. Compared with the actuators mentioned above, the linear electromagnetic microactuator can provide large displacement, and high precision control in a small volume with a low driving voltage [15,16,17,18,19]. The linear microactuator also has higher control accuracy and faster response [20,21,22,23]. However, for linear electromagnetic microactuators, the planar microcoil and the permanent magnet were fabricated on two separate wafers, and the prototype was formed using a bonding process. In current study, although the volume was smaller, the fabrication process was simplified, and the characteristics was obviously improved, the linear microactuator’s high integrity, large displacement and high precision can be improved further.

## 2. Structural Design of the Linear Magnetic Microactuator

**Figure 1:**
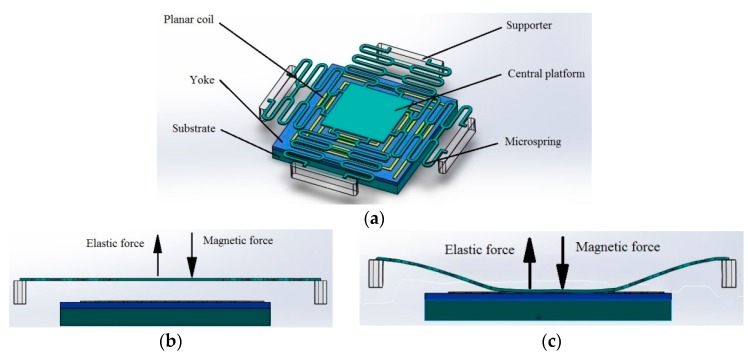
The structure and working mechanism of the microactuator. (**a**) Structure; (**b**) Open state; (**c**) Closed state.

The linear magnetic microactuator consists of a planar microcoil, a supporter and a microspring, as shown in Figure 1a. The microcoil can provide bidirectional electromagnetic force, which can increase or decrease the magnetic force in combination with the external permanent magnetic force on the microspring. The dimension and material of the microactuator is shown in Table 1. The microactuator is usually in either open or closed state, and switching between the two states is achieved by the balance between the magnetic force and the elastic force. If the microactuator is in the open state, the microspring stays flat, as shown in Figure 1b. When a positive current is fed into the microcoil, an electromagnetic force is generated, and the magnetic force on the microspring increases. When the magnetic force is larger than the elastic force, the microspring will be attracted down to the bottom, and the microactuator is switched to closed state. At the same time, the current is decreased, and the microactuator remains in closed state because of the permanent magnetic force, as shown in Figure 1c. This means the microactuator can work in a closed state without current, which reduces its power consumption greatly. When a negative current is fed into the planar microcoil, a negative electromagnetic force is generated, and the magnetic force is reduced. When the magnetic force is smaller than elastic force, the microspring will be pushed up into the open position as a result of the elastic force, as shown in Figure 1a.

**Figure 2:**
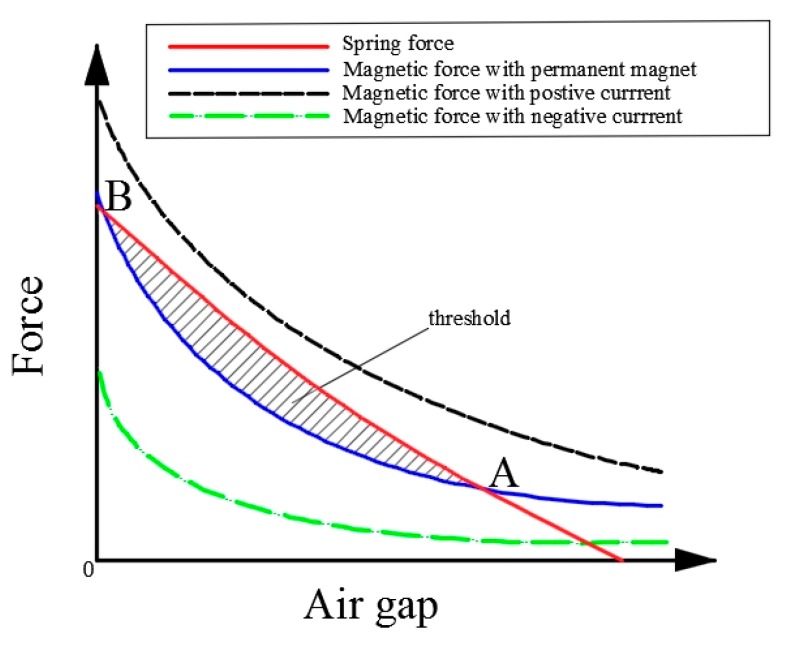
The relationship between the electromagnetic force, the elastic force and the air gap.

The working principle of the microactuator is dependent on the matching between the magnetic force and the elastic force, as shown in Figure 2. There are two intersections of the magnetic force curve and the elastic curve; that is, they enclose the working threshold. By changing the direction of the current in the microcoil, the electromagnetic force becomes positive or negative, the magnetic force is increased or decreased, and two stable states can be obtained. It is assumed that the initial state corresponds to the disconnected state, that is, point A in Figure 2, which indicates the first stable state. When a positive current is applied, the magnetic force increases, which becomes larger than the elastic force. Then the microactuator is closed at B point indicated in Figure 2. In this state, the closing magnetic force is provided only by external permanent magnet without extra power consumption. When the reverse current is applied, the electromagnetic force is gradually reduced. The microactuator will then return to the disconnected state.

## 3. Topology Design and Simulation of the Microspring

**Figure 3:**
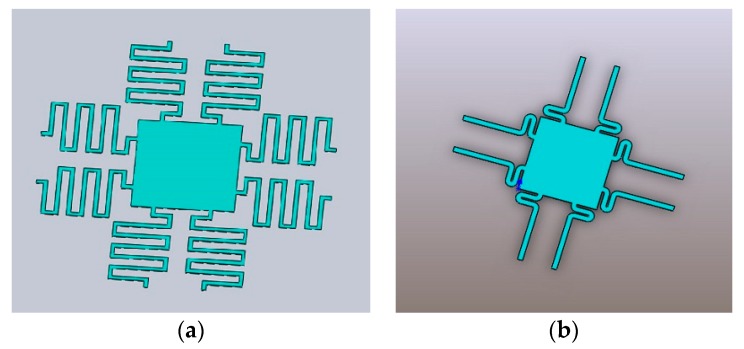

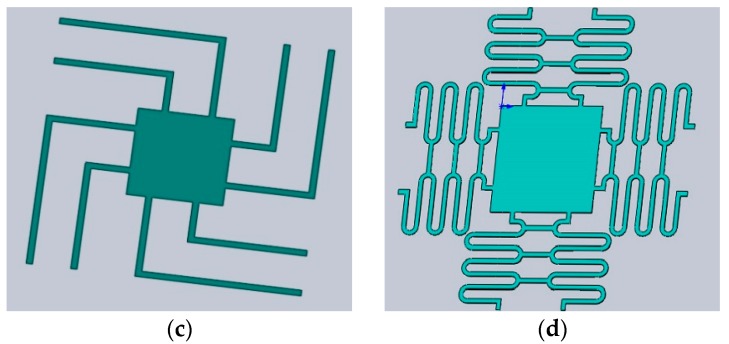
Four kinds of cantilever beam structure. (**a**) U-type; (**b**) N-type; (**c**) L-type; (**d**) W-type.

The switching of states in the microactuator is obtained by the elastic deformation of the micro spring, so the spring’s linearity has a great influence on the working mechanism. In order to ensure the precise movement of microactuator, the microspring needs to provide a compliant linear motion. Through our previous experimental research combined with other researchers’ experimental conclusions in [24,25], it is found that the rectangular cantilever beam in the traditional process can provide both translation motion and rotation error, which causes a torsion pendulum and affects the accuracy. Thus, a symmetrical structure can reduce the rotation error and achieve precise control of pure translational motion. As a result, a spring with four topology structures was designed, as shown in Figure 3. In order to analyze the elastic force, ANSYS (12.0 version, Canonsburg, PA, USA) was used to carry out a three-dimensional simulation experiment of our MEMS microactuator.

**Figure 4:**
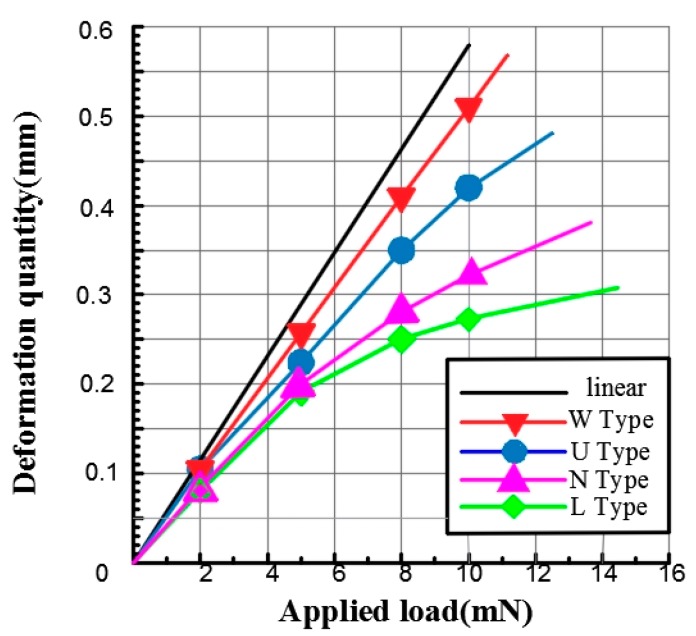
Nonlinear simulation curves of four kinds of structural springs.

According to the traditional mechanism, the elastic force is proportional to the deformation. However, in natural conditions, nonlinear phenomena are common. The nonlinearity of the structure will lead to a nonlinear relationship between the external load and the strain generated by the structure, which leads to the microactuator being uncontrollable. Therefore, ANSYS static simulation of each spring model was carried out on four different topology structures, and the load-carrying capacity increased from 2 to 15 mN. The maximum spring deformation value was obtained under the nonlinear calculation conditions at each load, and compared with the linear value as the characterization of the nonlinear degree. As shown in Figure 4, the abscissa is the load value, the ordinate is the spring deformation, and the thick black line is the linear result curve. By comparing the nonlinear deformation of four cantilever structures under different load forces, it can be obtained that different structure has different linearization.

The nonlinear curves of the four springs with the same linear stiffness and different plane geometry gradually deviate from the linear deformation curve, and the offset increases sequentially. The nonlinear results corresponding to the U and W-type springs are close to the linear results, and the elastic coefficient curves are close to the straight line, which shows that the nonlinear degree of the two shapes and sizes of the springs is low, and the d spring is the lowest. The deformation of W spring under an 8 mN load is close to that of a-type spring, but the deformation increases after a 10 mN load. It can be seen that for different topological structures, different nonlinear characteristics will be generated under nonlinear conditions. 

## 4. Simulation of the Bistable Mechanism of the Microactuator

The above results provided a basis for further reducing the nonlinear characteristics of springs under large deformation conditions through topology design. It is also proved that the structure topology optimization design method using ANSYS can effectively analyze the influence of the nonlinear degree of the microspring from the geometric nonlinear angle, and reduce the influence of the nonlinearity on the bistability of the device through structural topology optimization.

**Figure 5:**
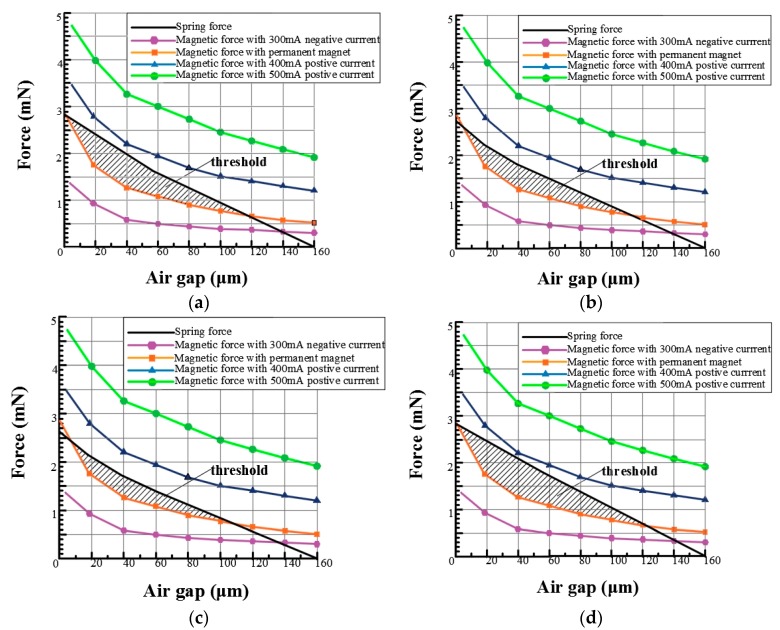
Matching diagram of elastic force and magnetic force for microsprings with four topology structures. (**a**) U-type; (**b**) N-type; (**c**) L-type; (**d**) W-type.

ANSYS was used to conduct the magnetic simulation of the whole magnetic circuit structure. Due to the symmetry of the whole structure, only a quarter of the structure needed to be modeled and analyzed. First, the overall parameters of the structure were determined: The number of the coil is 30 turns; the relative permeability of the permanent magnet is 50,000; the coercive force is 1 × 10^5^ A/m and the air gap size is 160 μm. Under the condition of these constant structural parameters, analysis of the bistable mechanism of microactuators with four different topology structures of the microspring was carried out with 300 mA negative current, 400 mA positive current, 500 mA positive current, and permanent magnet force. The results are shown in Figure 5.

When a 300 mA negative current is applied, the resultant electromagnetic force is less than the spring force, and the microactuator is returned to its original state. When a 400 mA and 500 mA positive current are applied, the resultant electromagnetic force is greater than the spring force, and the microactuator is closed. In addition, the spring force curve has two intersection points with the magnetic force produced by the permanent magnet, which indicates the bistable mechanism of microactuator.

In order to compare the threshold formed by the two curves for the four topological structures, the curve-fitting method is used to fit the data of the curves generated by permanent magnet and the four curves. First, the polynomial fitting method is applied to the electromagnetic force curve. Assuming that the air gap *x* is an independent variable, the electromagnetic force *F* is a dependent variable. In order to ensure the accuracy of the curve, 5th-order polynomials are used to fit the data. It can be obtained that:

(1) $$F=p_{1}\times x^{5}+p_{2}\times x^{4}+p_{3}\times x^{3}+p_{4}\times x^{2}+p_{5}\times x+p_{6}\;$$

It can be seen that the determinable coefficient of the fitting curve *R*-square is 0.9996 and the correction factor adjusted *R*-square is 0.9989, so the fitting accuracy is higher and the error is smaller, and it can be used for the calculation of the threshold. The results of the polynomial are in Table 2.

Secondly, four groups of elastic force curves were fitted: Elastic curve of the U-type spring:

(2) $$F_{a}=2.5e^{-5}\times x^{2}-0.0215x+2.8\;$$

Elastic curve of the N-type spring:

(3) $$F_{b}=3.081e^{-5}\times x^{2}-0.0212x+2.604\;$$

Elastic curve of L-type spring:

(4) $$F_{c}=3.556e^{-5}\times x^{2}-0.0219x+2.604\;$$

Elastic curve of W-type spring:

(5) $$F_{d}=-0.0175x+2.8\;$$

The integral method was used to calculate the threshold area of the force curve and the electromagnetic force curve. The solution is shown in Table 3.

From the calculated threshold area, it can be obtained that the threshold area is smaller with the stronger nonlinearity. The threshold area of N-type (D_b_) and L-type (D_c_) springs with the worst linearity is 23.45 and 22.74 respectively, which is obviously smaller than the threshold area formed by the U-type (D_a_) and W-type (D_d_) springs. In the matching curve between U-type and W-type springs and electromagnetic force, the degree of similarity of the two curves is similar when the air gap spacing is less than 60 µm. When the gap spacing is greater than 60 µm, it can be seen that the nonlinear degree of the elastic resilience curve of the U-type spring is enhanced, which leads to the reduction of the threshold area. Therefore, the threshold area of the W spring structure is larger, which indicates better reliability of the bistable mechanism.

**Figure 6:**
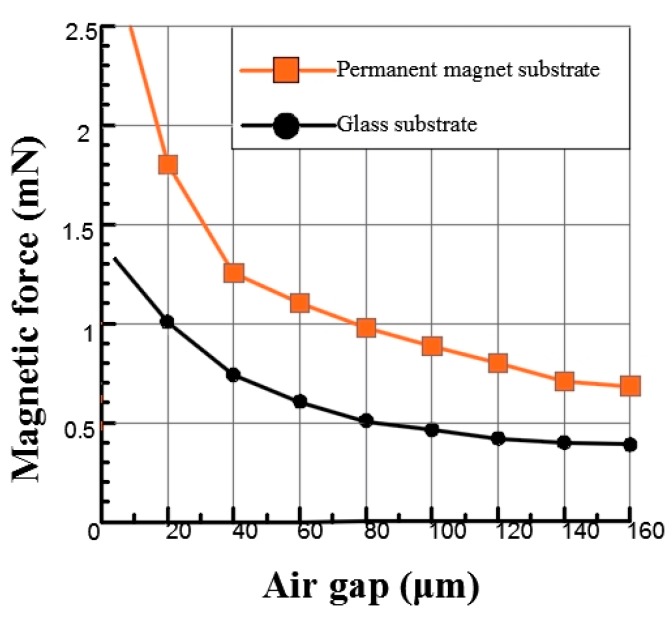
Variation of magnetic force with current on different substrates.

In addition, the electromagnetic force analysis of the above simulation was based on the analysis of the permanent magnet substrate. By analyzing the electromagnetic force produced by different substrate materials, the effects of different substrate materials on the size of the electromagnetic force were analyzed in the case of the same overall structure. With the same 300 mA current and overall structure size, two materials of permanent magnet and glass were used as the substrates of the microactuator, as shown in Figure 6. Because the permanent magnetic material can generate extra magnetic force on the microspring, a larger magnetic force can be produced for the same current by using a permanent magnet substrate to ensure a higher driving speed. 

Finally, through the simulation and analysis, it can be seen that a permanent magnet substrate and the W-type microspring can reduce the influence of nonlinearity on the bistable mechanism at 300 mA of current. Thus, the response speed and working stability of the microactuator is improved. 

## 5. Fabrication and Testing of the Bistable Microactuator

**Figure 7:**
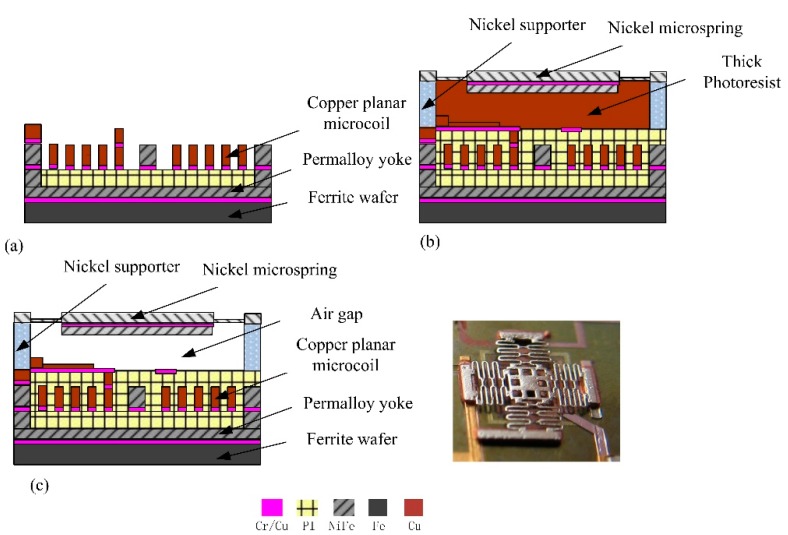
The fabrication process and the prototype.

The bistable magnetic microactuator was fabricated based on the non-silicon micro-micromachining process on a single wafer. The fabrication is described as follows: (a) The chromium/copper seed layer was sputtered on to the ferrite wafer, and then the photoresist was spin coated, followed by electroplating the permalloy yoke and copper planar microcoil. (b) After forming polyimide as an insulation layer for the planar microcoil, the photoresist was coated on two layers thick, then heat treating as sacrificial layer for the air space between the microactuator and microspring. The supporter was then electroplated, followed by the microspring. (c) Last, the thick photoresist and Cr/Cu seed layer were etched smoothly layer by layer, then the suspended structure was released and the spring could be moved in a space as shown in Figure 7.

**Figure 8:**
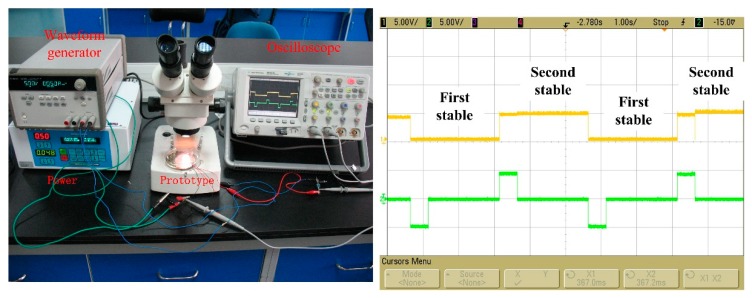
Bistable mechanism of the prototype.

The bistable mechanism testing system was established, and 5 V, generated by a B&K 2706 power amplifier (Agilent 6813B, Agilent company, Santa Clara, CA, USA) incorporated with a GW waveform generator (GFG-8016G, RIGOL, Beijing, China), was applied to the microcoil. The results were observed by the oscilloscope (Agilent MSO6034, Agilent company). By comparing the input signal (lower level) and output signal (upper level), the bistable mechanism could be observed as shown in Figure 8. The microactuator is in the first stable state, then, when the positive current is fed into the coil, the microactuator is switched into the second stable state. When the current was reduced, the microactuator could stay in the second stable state without power consumption until the next switching current. The difference between the driving voltage and switching voltage at the higher level means a response time of 0.96 ms. The bistable mechanism verified the reliability of the design and simulation described above.

## 6. Conclusions

This paper presents a linear microactuator, which has high response, large displacement and high precision for use in portable electronic equipment and microsatellite high resolution remote sensing technology. The linear microactuator comprises a planar microcoil, a supporter and a microspring. In order to optimize the linearity of the microactuator, microsprings with four topology structures were simulated and analyzed. By comparing the simulation results, the best linear spring, d-type, could improve the threshold of the bistable mechanism. The redundancy of the fabrication process of the linear microactuator is increased, which has a positive influence on the precision of the microactuator. The linear microactuator was fabricated based on nonsilicon process, and the bistable mechanism of the prototype was tested. The results showed that the prototype could realize a bistable mechanism with a response time of 0.96 ms, which verified the reliability of the topology design and simulation.

## Figures and Tables

**Table 1:** Properties of the microactuator.

Part	Material	Value
Central Platform	Permalloy	1 mm × 1 mm × 15 µm
Planar Microcoil	Copper	2.5 mm
Microspring	Nickel	3 mm × 3 mm × 12 µm
Yoke	Permalloy	2.8 mm × 2.8 mm × 50 µm
Substrate	Ferrite	2.8 mm × 2.8 mm × 200 µm
Supporter	Nickel	1.2 mm × 0.2 mm × 160 µm

**Table 2:** Results of the polynomial.

*p*_1_ = −1.551 × e^−10^	*p*_2_ = 8.225 × e^−8^
*p*_3_ = −1.691 × e^−5^	*p*_4_ = 0.0017
*p*_5_ = −0.08869	*p*_6_ = 3.003

**Table 3:** Threshold area of four types of microactuator spring.

Type	Threshold Area
D_a_	40.2
D_b_	23.45
D_c_	22.74
D_d_	53.48
